# Non-dissociative structural transitions of the Watson-Crick and reverse Watson-Crick А·Т DNA base pairs into the Hoogsteen and reverse Hoogsteen forms

**DOI:** 10.1038/s41598-018-28636-y

**Published:** 2018-07-10

**Authors:** Ol’ha O. Brovarets’, Kostiantyn S. Tsiupa, Dmytro M. Hovorun

**Affiliations:** 1 Department of Molecular and Quantum Biophysics, Institute of Molecular Biology and Genetics, National Academy of Sciences of Ukraine, 150 Akademika Zabolotnoho Str., 03680 Kyiv, Ukraine; 2 Department of Molecular Biotechnology and Bioinformatics, Institute of High Technologies, Taras Shevchenko National University of Kyiv, 2-h Akademika Hlushkova Ave., 03022 Kyiv, Ukraine

## Abstract

In this study it was theoretically shown that discovered by us recently (Brovarets’ *et al*., *Frontiers in Chemistry*, 2018, 6:8; doi: 10.3389/fchem.2018.00008) high-energetical, significantly non-planar (symmetry C_1_), short-lived wobbled conformers of the classical Watson-Crick А·Т(WC), reverse Watson-Crick А·Т(rWC), Hoogsteen А·Т(Н) and reverse Hoogsteen А·Т(rН) DNA base pairs are the intermediates of their pairwise А∙Т(WC)/А∙Т(rWC) ↔ А∙Т(H)/А∙Т(rH) conformational transformations. These transitions do not require for their realization the energy-consumable anisotropic rotation of the amino group of A around the exocyclic C6-N6 bond. They are controlled by the non-planar transition states with quasi-orthogonal geometry (symmetry C_1_) joined by the single intermolecular (Т)N3H···N6(А) H-bond (~4 kcal∙mol^−1^). The Gibbs free energies of activation for these non-dissociative, dipole-active conformational transitions consist 7.33 and 7.81 kcal∙mol^−1^, accordingly. Quantum-mechanical (QM) calculations in combination with Bader’s quantum theory of “Atoms in Molecules” (QTAIM) have been performed at the MP2/aug-cc-pVDZ//B3LYP/6-311++G(d,p) level of QM theory in the continuum with ε = 4 under normal conditions.

## Introduction

Spontaneous transition of the DNA base pairs from the Watson-Crick (WC) to Hoogsteen (H) configuration and *vice versa* is one of the functionally-important physico-chemical properties of DNA^1–9^. It was shown by NMR methods^1–5^ that Watson-Crick ↔ Hoogsteen breathing in DNA duplex containing A∙T rich region occurs *via* the switching of the Watson-Crick DNA base pair (bp) from the *anti-* to *syn-*conformation with the probability ~10^−2^ and represents one of the pathways for the reaction of formaldehyde with DNA^10^. Thorough calculations by the method of molecular dynamics indicate that А·Т(WC) ↔ А·Т(Н) transitions of actually bps and *anti* *↔* *syn* transitions of the A around the glycosidic bond are closely correlated processes, for which Gibbs free energy of activation is 10–11 kcal∙mol^−1^ under normal conditions^8^.

Based on analysis of the microstructural nature of these transitions, it is quite logical to connect it with the analogical properties of the isolated DNA bps^11–13^. Comprehensive analysis of the current literature data showed that the nature of these biologically-important processes has not been investigated at all. Currently in the literature there is only one single theoretical work devoted to the study of the *anti* ↔ *syn* non-dissociative transitions in irregular pairs of nucleotide bases that do not have an exocyclic amino group in its composition^14^.

Recently, we have theoretically revealed novel high-energetic, significantly non-planar (symmetry C_1_), short-lived wobbled (w) conformers – А·Т(w_WC_), А·Т(w_rWC_), А·Т(w_Н_) and А·Т(w_rН_) for each of the four classical А·Т(WC) DNA bps – Watson-Crick А·Т(WC), reverse Watson-Crick А·Т(rWC), Hoogsteen А·Т(Н) and reverse Hoogsteen А·Т(rН)^11^. It is known from the literature data, that these bps joined by different H-bonds are formed due to the rotation of the DNA base relative to the other on 180° around: the (A)N1–N3(T) axis for the reverse Watson-Crick А·Т(rWC) or in other terms Donohue DNA bp^15–23^; the (A)C9′-N9 axis for the Hoogsteen A·T(H) bp^1–30^ and the (A)N7–N3(T) axis for the reverse Hoogsteen A·T(rH) or in other terms Haschemeyer–Sobell bp^31–34^.

It was found that revealed А·Т(w_WC_), А·Т(w_rWC_), А·Т(w_Н_) and А·Т(w_rН_) conformers have essentially non-planar structure joined by the two anti-parallel N6H/N6H′···O4/O2 and N3H···N6 H-bonds (the N6H′ chemical bond has *trans-*orientation relative to the N1C6 chemical bond of A). These specific intermolecular contacts involve pyramidalized A amino group, acting simultaneously as an acceptor and a donor of the H-bonding. The transition states (TSs) – TS_А·Т(WC)↔А·Т(wWC)_, TS_А·Т(rWC)↔А·Т(wrWC)_, TS_А·Т(Н)↔А·Т(wН)_ and TS_А·Т(rН)↔А·Т(wrН)_ – of the dipole-active conformational transformations of the basic, plane-symmetric state of the classical А·Т DNA bps into the high-energetic, essentially non-planar wobbled bps and *vice versa* possess wobble structures (symmetry C_1_) and are joined by the N6H/N6H′···O4/O2 and N3H···N6 H-bonds. The А·Т(w_WC_), А·Т(w_rWC_), А·Т(w_Н_) and А·Т(w_rН_) conformers was found to be dynamically stable structures with short lifetime τ = (1.4–3.9) ps. It was assumed that these conformational transitions are directly related to the thermally-driven fluctuational behavior of DNA – pre-melting and breathing^6,7^.

In this work it was established for the first time that just-mentioned novel conformers А·Т(w_WC_), А·Т(w_rWC_), А·Т(w_Н_) and А·Т(w_rН_) control the А·Т(w_WC_)/А·Т(w_rWC_) ↔ А·Т(w_Н_)/А·Т(w_rН_) conformational transitions. Moreover, in view of the recently discovered conformational transitions for the classical A·T DNA bps - А·Т(WC) ↔ А·Т(w_WC_), А·Т(rWC) ↔ А·Т(w_rWC_), А·Т(Н) ↔ А·Т(w_Н_) and А·Т(rН) ↔ А·Т(w_rН_)^11^, they are also intermediates of the biologically-important А·Т(WC)/А·Т(rWC) ↔ А·Т(Н)/А·Т(rН) conformational transitions.

Energetically favorable mechanism of the conformational pairwise transformation of the intermediates А∙Т(w_WC_) ↔ А∙Т(w_H_) and А∙Т(w_rWC_) ↔ А∙Т(w_rH_), and together with them conformational transition of the А∙Т DNA bps – А∙Т(WC)/А∙Т(rWC) ↔ А∙Т(H)/А∙Т(rH), does not require for their realization the rotation of the amino group of A around the exocyclic C6N6 bond^35^.

In this case conformational transformations are controlled by the soft, non-planar TSs, stabilized by the participation of the single intermolecular (Т)N3H···N6(А) H-bond between the imino group of T and pyramidilized amino group of A. The Gibbs free energies of activation for these non-dissociative, dipole-active conformational transitions consist 7.33 and 7.81 kcal∙mol^−1^, accordingly.

Two other mechanisms – the А∙Т(w_WC_) ↔ А∙Т(w_H_) and А∙Т(w_rWC_) ↔ А∙Т(w_rH_) – are realized *via* the anisotropic rotation of the amino group of A (together with T interacting with A through two intermolecular antiparallel (A)N6H/N6H′···O4/O2(T) and (T)N3H···N6(A) H-bonds) around the exocyclic C6N6 bond. In TSs of these conformational transitions the pyramidality of the amino group of A significantly increases: this causes increase of the energy of the N3H···N6 H-bond and decrease of the energy of the intermolecular N6H/N6H′···O4/O2 H-bond. The transitions states of these reactions – TS^cys^_А·Т(wWC)↔А·Т(wН)_, TS^trans^_А·Т(wWC)↔А·Т(wН)_ and TS^cys^_А·Т(wrWC)↔А·Т(wrН)_, TS^trans^_А·Т(wrWC)↔А·Т(wrН)_ – have close energy in corresponding conformational transformations (14.9 and 15.0 kcal∙mol^−1^, accordingly). Thus, these TSs of the mutual conformational transformation of the wobble intermediates – А∙Т(w_WC_) ↔ А∙Т(w_H_) and А∙Т(w_rWC_) ↔ А∙Т(w_rH_) of the classical А∙Т DNA bps – А∙Т(WC)/А∙Т(rWC) ↔ А∙Т(H)/А∙Т(rH) – determine their conformational transformations.

## Computational Methods

We have calculated geometries of the basic and high-energetic conformers and transition states (TSs) of their mutual conformational transformations together with their harmonic vibrational frequencies at the B3LYP/6–311++G(d,p) level of theory^36–40^, using Gaussian’09 package^41^, in the continuum with ε = 4, which is typical for the processes in real biological complexes and taking into account the structural and functional characteristics of the bases in the duplex DNA and at the same time satisfactorily reflecting the environment in the essentially hydrophobic base-pair recognition pocket of the high-fidelity DNA-polymerase^42–66^. Considered level of theory has been successfully applied for the calculations of the similar tasks and systems^47–55^. A scaling factor of 0.9668^55–61^ has been used in order to correct the harmonic frequencies of all bps and TSs of the transitions between them. The local minima or TSs, localized by Synchronous Transit-guided Quasi-Newton method^62^, have been appointed to the complexes on the potential energy landscape containing any or one imaginary frequency in their vibrational spectra, accordingly. We used TS theory in order to estimate the activation barriers of the conformational transformations^63^. Electronic energy calculations have been performed at the single point at the MP2/aug-cc-pVDZ level of theory^67,68^.

The Gibbs free energy G for all structures has been received at the MP2/6-311++G(2df,pd) level of theory by the formula:

1 $${\rm{G}}={{\rm{E}}}_{{\rm{el}}}+{{\rm{E}}}_{{\rm{corr}}},$$

where E_el_ – electronic energy, while E_corr_ – thermal correction.

The electronic energies of interaction ∆E_int_ have been obtained at the MP2/6-311++G(2df,pd) level of theory as a difference between the BSSE-corrected^69–72^ electronic energy of the bp and electronic energies of the isolated bases.

Bader’s quantum theory of Atoms in Molecules (QTAIM)^73–78^ has been applied for the analysis of the electron density distribution by AIMAll program package^79^, using wave functions calculated at the B3LYP/6-311++G(d,p) level of theory. We considered the presence of the (3, −1) bond critical point (BCP), a bond path between the donor and acceptor of the intermolecular contact and positive value of the Laplacian at this BCP (Δρ > 0) as criteria for the existence of the H-bond or attractive van der Waals contact formation^73–84^.

The energies of the attractive van der Waals contacts^85,86^ in TSs of the conformational transitions have been estimated by the Espinosa-Molins-Lecomte (EML) formula^87,88^:

2 $$E={\rm{0.5}}\cdot {\rm{V}}({\rm{r}}),$$

where V(r) – value of a local potential energy at the (3, −1) BCP.

The energies of the conventional AH···B H-bonds have been calculated by the Iogansen’s formula^89^:

3 $${E}_{AH\cdot \cdot \cdot B}=0.33\cdot \sqrt{{\rm{\Delta }}\nu -40},$$

where Δν – frequency shift of the stretching mode of the H-bonded AH group involved in the AH···B H-bond relatively the unbound group. We applied the partial deuteration in order to avoid the effect of vibrational resonances^90,91^.

In this study the numeration for the DNA bases is generally accepted^92^.

In this study we have provided investigations at the basic, but sufficient level of the isolated H-bonded pairs of nucleotide bases, that adequately simulates the processes in real biological systems^93–95^, in particular in the base-pair recognition pocket of the high-fidelity DNA-polymerase^42–46^. At this, we have relied on the experience received in the previous works^11,96–98^ on the related topic and systems, in which the negligibly small impact of the stacking and sugar-phosphate backbone on the tautomerisation processes has been shown.

## Results and Their Discussion

In our previous paper^11^ we have succeed to establish in the classical А∙Т DNA bps with C_s_ symmetry – Watson-Crick (WC), reverse Watson-Crick А·Т(rWC), Hoogsteen А·Т(Н) and reverse Hoogsteen А·Т(rН) DNA bps – novel high-energetic, dynamically-stable, mirror-symmetrical А∙Т(w_WC_)_R,L_, А∙Т(w_H_)_R,L_, А∙Т(w_rWC_)_R,L_ and А∙Т(w_rH_)_R,L_ conformational states. Their distinguished feature independently of the pair, in which they are realized, is significantly non-planar structure (С_1_ symmetry), caused by the pyramidal structure of the ≥C6N6H_2_ amino fragment of the A DNA base, in which the amino group acts simultaneously as a donor and an acceptor of the specific intermolecular interaction with T through the two (Т)N3H···N6(A) and (A)N6H/N6H′···O4/O2(T) H-bonds. Each of the four А∙Т Watson-Crick DNA bps transfers into the aforementioned conformer *via* two mirror-symmetric pathways through the TS_А∙Т(WC)↔А∙Т(wWC)R,L_, TS_А∙Т(rWC)↔А∙Т(wrWC)R,L_, TS_А∙Т(H)↔А∙Т(wrH)R,L_ and TS_А∙Т(rH)↔А∙Т(wrH)R,L_ (C_1_ symmetry). At this, the structures, which names differ from each other only by the subscripts R and L, are mirror-symmetrical, that is enantiomers. It is well known that enantiomers have identical scalar physico-chemical characteristics and differ only by the direction of the dipole moment.

Let analyze the biological significance of these non-usual conformers of the classical А∙Т DNA bps.

**Figure 1:**
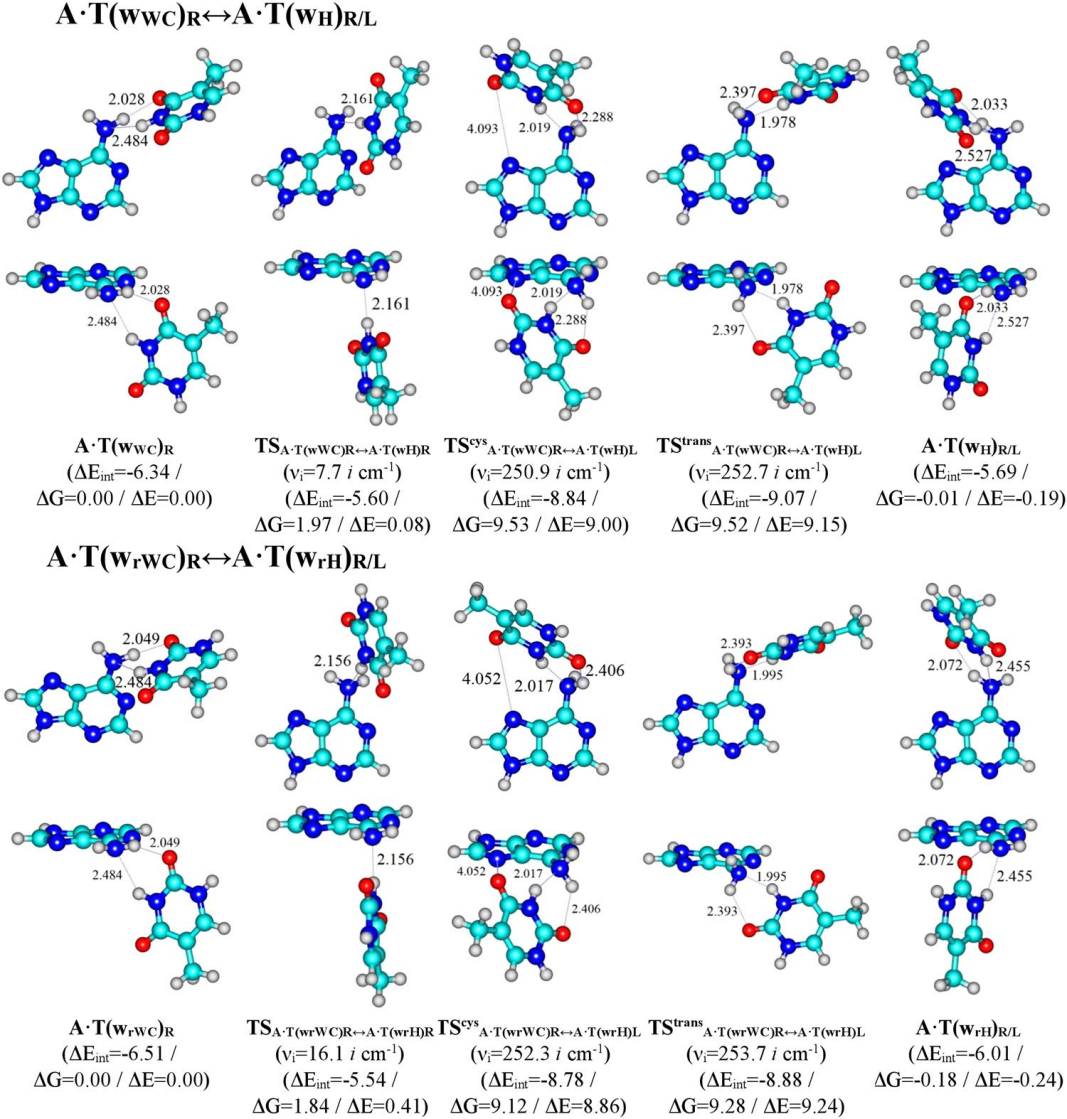
Geometrical structures of the stationary points on the reaction pathways of the discovered conformational transitions of the four biologically important А·Т DNA bps. Electronic energies of the interaction ΔE_int_ (MP2/6-311++G(2df,pd)//B3LYP/6-311++G(d,p) level of theory, in kcal∙mol^−1^), relative Gibbs free energies ∆G and electronic energies ∆E (in kcal∙mol^−1^), imaginary frequencies *ν*_*i*_ at the TSs of the conformational transitions (MP2/aug-cc-pVDZ//B3LYP/6-311++G(d,p) level of theory in the continuum with ε = 4 at T = 298.15 К) are presented below complexes in brackets. Dotted lines indicate AH···B H-bonds and attractive A···B van der Waals contacts – their lengths are presented in angstroms (for their more detailed physico-chemical characteristics see Table 2); carbon atoms are in light-blue, nitrogen – in dark-blue, hydrogen – in grey and oxygen – in red. Exclusively enantiomers of one type are presented.

In this context it was fixed important result – these conformers are responsible for the two different WC/rWC ↔ H/rH mechanisms of the non-dissociative conformational transformation of the А∙Т DNA bps (Fig. 1, Tables 1–3).

**Table 1:** Energetic characteristics (in kcal∙mol^−1^) of the discovered conformational transitions of the four biologically important А·Т DNA bps obtained at the MP2/aug-cc-pVDZ//B3LYP/6-311++G(d,p) level of theory in the continuum with ε = 4 (see Fig. 1).

Conformational transition	ν_i_^a^	∆G^b^	∆E^c^	∆∆G_TS_^d^	∆∆E_TS_^e^	∆∆G^f^	∆∆E^g^
А·Т(w_WC_)_R,L_ ↔ А·Т(w_Н_)_R,L_	7.7	−0.01	−0.19	1.97	0.08	1.98	0.28
А·Т(w_WC_)_R,L_ $\mathop{\longleftrightarrow }\limits^{cys}$ А·Т(w_Н_)_L,R_	250.9	−0.01	−0.19	9.53	9.00	9.54	9.19
А·Т(w_rWC_)_R,L_ $\mathop{\longleftrightarrow }\limits^{trans}$ А·Т(w_rН_)_L,R_	252.7	−0.01	−0.19	9.52	9.15	9.53	9.34
А·Т(w_rWC_)_R,L_ ↔ А·Т(w_rН_)_R,L_	16.1	−0.18	−0.24	1.84	0.41	2.02	0.64
А·Т(w_rWC_)_R,L_ $\mathop{\longleftrightarrow }\limits^{cys}$ А·Т(w_rН_)_L,R_	252.3	−0.18	−0.24	9.12	8.86	9.30	9.09
А·Т(w_rWC_)_R,L_ $\mathop{\longleftrightarrow }\limits^{trans}$ А·Т(w_rН_)_L,R_	253.7	−0.18	−0.24	9.28	9.24	9.46	9.48

^a^Imaginary frequency at the TS of the conformational transition, cm^−1^. ^b^The Gibbs free energy of the product relatively the reactant of the conformational transition (T = 298.15 K). ^c^The electronic energy of the product relatively the reactant of the conformational transition. ^d^The Gibbs free energy barrier for the forward conformational transition. ^e^The electronic energy barrier for the forward conformational transition. ^f^The Gibbs free energy barrier for the reverse conformational transition. ^g^The electronic energy barrier for the reverse conformational transition.

**Table 2:** Electron-topological, geometrical and energetic characteristics of the intermolecular specific contacts in the investigated conformers of the А·Т DNA bps and TSs of their conformational transformations obtained at the B3LYP/6-311++G(d,p) level of theory (ε = 4) (see Fig. 1).

Complex	AH···B H-bond/A···B vdW contact	*ρ* ^a^	Δ*ρ*^b^	100·*ε*^c^	d_*A∙∙∙B*_^d^	d_*H∙∙∙B*_^e^	∠AH∙∙∙B^f^	E_*AH···B*_/°E_*A···B*_^g^	μ^h^
А·Т(w_WC_)_R,L_^11^	N6H∙∙∙O4	0.022	0.076	2.10	2.988	2.028	156.2	4.11	3.97
	N3H∙∙∙N6	0.010	0.030	31.69	3.337	2.484	141.1	1.75	
TS_А·Т(wWC)R,L↔А·Т(wН)R,L_	N3H∙∙∙N6	0.019	0.055	3.09	3.184	2.161	180.0	4.02	5.20
TS^cys^_А·Т(wWC)R,L↔А·Т(wН)L,R_	N6H∙∙∙O4	0.014	0.045	11.55	3.083	2.288	133.5	1.81	5.25
	N3H∙∙∙N6	0.026	0.076	3.24	2.976	2.019	153.3	5.52	
	O2∙∙∙N7	0.001	0.005	83.95	4.093	**—**	**—**	0.17*	
TS^trans^_А·Т(wWC)R,L↔А·Т(wН)L,R_	N6H′∙∙∙O4	0.011	0.037	18.97	3.134	2.397	128.3	1.29	3.32
	N3H∙∙∙N6	0.029	0.081	2.33	2.953	1.978	156.4	5.88	
А·Т(w_Н_)_R,L_^11^	N6H′∙∙∙O4	0.021	0.075	2.64	2.983	2.033	154.4	4.01	8.29
	N3H∙∙∙N6	0.009	0.028	34.33	3.370	2.527	140.1	1.55	
А·Т(w_rWC_)_R,L_^11^	N6H∙∙∙O2	0.020	0.072	1.98	3.000	2.049	154.6	3.85	3.71
	N3H∙∙∙N6	0.010	0.030	26.08	3.332	2.484	140.6	1.81	
TS_А·Т(wrWC)R,L↔А·Т(wrН)R,L_	N3H∙∙∙N6	0.019	0.056	3.16	3.157	2.156	165.7	3.94	5.43
TS^cys^_А·Т(wrWC)R,L↔А·Т(wrН)L,R_	N6H∙∙∙O2	0.011	0.036	19.20	3.143	2.406	128.4	1.05	4.88
	N3H∙∙∙N6	0.027	0.076	2.09	2.979	2.017	154.4	5.54	
	O4∙∙∙N7	0.001	0.005	235.50	4.052	—	—	0.19*	
TS^trans^_А·Т(wrWC)R,L↔А·Т(wrН)L,R_	N6H′∙∙∙O2	0.011	0.036	17.71	3.137	2.393	129.0	1.21	5.20
	N3H∙∙∙N6	0.028	0.079	2.26	2.962	1.995	155.0	5.74	
А·Т(w_rН_)_R,L_^11^	N6H′∙∙∙O2	0.020	0.069	2.88	2.998	2.072	150.5	3.71	8.26
	N3H∙∙∙N6	0.010	0.032	21.42	3.308	2.455	141.1	1.55	

^a^The electron density at the (3, −1) BCP of the specific contact, a.u. ^b^The Laplacian of the electron density at the (3, −1) BCP of the specific contact, a.u. ^c^The ellipticity at the (3, −1) BCP of the specific contact. ^d^The distance between the A and B atoms of the specific contact, Å. ^e^The distance between the H and B atoms of the AH···B H-bond, Å. ^f^The H-bond angle, degree. ^g^Energy of the AH···B H-bond or attractive A···B van der Waals (vdW) contact, calculated by Iogansen’s or Espinose-Molins-Lecomte (marked with an asterisk) formulas, kcal∙mol^−1^. The dipole moment of the complex, D.

**Table 3:** Selected geometrical parameters, characterizing the non-planarity of the discovered conformers with wobble geometry of the four biologically important А·Т DNA bps and TSs of their conformational interconversions, obtained at the B3LYP/6-311++G(d,p) level of theory in the continuum with ε = 4.

Complex/Base	Dihedral angle, degree		
	C5C6N6H′	N1C6N6H	HN9N1H
А·Т(w_WC_)_R,L_	−13.8	14.9	−44.4
TS_А·Т(wWC)R,L↔А·Т(wН)R,L_	−22.8	22.1	−5.0
TS^cys^_А·Т(wWC)R,L↔А·Т(wН)L,R_	123.8	60.4	−49.1
TS^trans^_А·Т(wWC)R,L↔А·Т(wН)L,R_	57.3	120.3	−57.1
А·Т(w_Н_)_R,L_	−16.8	12.9	25.0
А·Т(w_rWC_)_R,L_	−14.2	15.4	99.4
TS_А·Т(wrWC)R,L↔А·Т(wrН)R,L_	−23.4	20.8	−130.3
TS^cys^_А·Т(wrWC)R,L↔А·Т(wrН)L,R_	124.0	60.3	63.9
TS^trans^_А·Т(wrWC)R,L↔А·Т(wrН)L,R_	57.4	120.8	−75.6
А·Т(w_rН_)_R,L_	−18.2	14.0	−88.0
A	−7.2	6.6	—
A^cys^	±57.9	∓122.1	—
A^trans^	±122.5	∓57.5	—

Note: Signs of the dihedral angles are presented exclusively for one type of enantiomers.

First of these conformational transformations, which are the most energetically favorable mechanisms, are controlled by the soft TS_А∙Т(wWC)R,L↔А∙Т(wH)R,L_ and TS_А∙Т(wrWC)R,L↔А∙Т(wrH)R,L_ (C_1_ symmetry) with low values of imaginary frequency (7.7 *i* and 16.1 *i* cm^−1^, accordingly). Both of them are joined by the one-single intermolecular (T)N3H···N6(A) H-bond (~4 kcal∙mol^−1^) between the imino group of T and pyramidilized amino group of A. In this case, conformational transformations of the А∙Т DNA bps are realized by the following non-dissociative scenario (each of them – by the mirror-symmetric pathways): А∙Т(WC) (0.00) ↔ TS_А∙Т(WC)↔А∙Т(wWC)R,L_ (7.13) ↔ А∙Т(w_WC_)_R,L_ (5.36)^11^ ↔ TS_А∙Т(wWC)R,L↔А∙Т(wH)R,L_ (7.33) ↔ А∙Т(w_H_)_R,L_ (5.35) ↔ TS_А∙Т(wH)R,L↔А∙Т(H)_ (7.24) ↔ А∙Т(H) (−0.44)^11^ and А∙Т(rWC) (0.00) ↔ TS_А∙Т(rWC)↔А∙Т(wrWC)R,L_ (7.26) ↔ А∙Т(w_rWC_)_R,L_ (5.97)^11^ ↔ TS_А∙Т(wrWC)R,L↔А∙Т(wrH)R,L_ (7.81) ↔ А∙Т(w_rH_)_R,L_ (5.79) ↔ TS_А∙Т(wrH)R,L↔А∙Т(rH)_ (7.41) ↔ А∙Т(rH) (−0.03)^11^. Notably, obtained energetic barriers are in good coincidence with the molecular-dynamic data for the А∙Т(WC) ↔ А∙Т(H) transition (10-11 kcal∙mol^−1^ under normal conditions^8^).

Herewith, some R structures transform into the other R structures, the same concerns L-structures. Saying in other words, pathways of these dipole-active conformational transformations are mirror-symmetric. In fact, the TS_А∙Т(wWC)R,L↔А∙Т(wH)R,L_ and TS_А∙Т(wrWC)R,L↔А∙Т(wrH)R,L_, which pairwise link the А∙Т(w_WC_)_R,L_ and А∙Т(w_H_)_R,L_, А∙Т(w_rWC_)_R,L_ and А∙Т(w_rH_)_R,L_ conformers, are transition states of the WC/rWC ↔ H/rH conformational transformations of the classical А∙Т DNA bps.

High-energetic mechanism of the WC/rWC ↔ H/rH conformational transitions of the А∙Т DNA bps is connected with anisotropic rotation of the amino group of A around the exocylic С6-N6 bond^35^ and is controlled by the TS^cys^_А∙Т(wWC)R,L↔А∙Т(wH)L,R_, TS^trans^_А∙Т(wWC)R,L↔А∙Т(wH)L,R_ and TS^cys^_А∙Т(wrWC)R,L↔А∙Т(wrH)L,R_, TS^trans^_А∙Т(wrWC)R,L↔А∙Т(wrH)L,R_, that have non-planar structure (С_1_ symmetry) and quite high values of the imaginary frequencies (~252 *i* cm^−1^). These TSs are joined by the two anti-parallel intermolecular (Т)N3H···N6(A) and (A)N6H/N6H′···O4/O2(T) H-bonds; notably, first of them is significantly stronger than the second one. The attractive O2···N7 and O4···N7 van der Waals contacts with weak energy (~0.18 kcal∙mol^−1^) also participate in the stabilization of the TS^cys^_А∙Т(wWC)R,L↔А∙Т(wH)L,R_ and TS^cys^_А∙Т(wrWC)R,L↔А∙Т(wrH)L,R_, accordingly.

In this case, the R structures transform into the L structures and *vice versa* and WC/rWC ↔ H/rH conformational transitions of the classical А∙Т DNA bps occur in such a case (each of them through two energetically and topologically non-equivalent ways):

А∙Т(WC) (0.00) ↔ TS_А∙Т(WC)↔А∙Т(wWC)R,L_ (7.13) ↔ А∙Т(w_WC_)_R,L_ (5.36)^11^ ↔ TS^cys^_А∙Т(wWC)R,L↔А∙Т(wH)L,R_ (14.89) ↔ А∙Т(w_H_)_L,R_ (5.35) ↔ TS_А∙Т(wH)L,R↔А∙Т(H)_ (7.24) ↔ А∙Т(H) (−0.44)^11^;

А∙Т(WC) (0.00) ↔ TS_А∙Т(WC)↔А∙Т(wWC)R,L_ (7.13) ↔ А∙Т(w_WC_)_R,L_ (5.36)^11^ ↔ TS^trans^_А∙Т(wWC)R,L↔А∙Т(wH)L,R_ (14.88) ↔ А∙Т(w_H_)_L,R_ (5.35) ↔ TS_А∙Т(wH)L,R↔А∙Т(H)_ (7.24) ↔ А∙Т(H) (−0.44)^11^;

А∙Т(rWC) (0.00) ↔ TS_А∙Т(rWC)↔А∙Т(wrWC)R,L_ (7.26) ↔ А∙Т(w_rWC_)_R,L_ (5.97)^11^ ↔ TS^cys^_А∙Т(wrWC)R,L↔А∙Т(wrH)L,R_ (15.01) ↔ А∙Т(w_rH_)_L,R_ (5.79) ↔ TS_А∙Т(wrH)L,R↔А∙Т(rH)_ (7.41) ↔ А∙Т(rH) (−0.03)^11^ and

А∙Т(rWC) (0.00) ↔ TS_А∙Т(rWC)↔А∙Т(wrWC)R,L_ (7.26) ↔ А∙Т(w_rWC_)_R,L_ (5.97)^11^ ↔ TS^trans^_А∙Т(wrWC)R,L↔А∙Т(wrH)L,R_ (15.00) ↔ А∙Т(w_rH_)_L,R_ (5.79) ↔ TS_А∙Т(wrH)L,R↔А∙Т(rH)_ (7.41) ↔ А∙Т(rH) (−0.03)^11^ (relative Gibbs free energy is presented after each structure in brackets at the MP2/aug-cc-pVDZ//B3LYP/6-311++G(d,p) level of QM theory in the continuum with ε = 4 under normal conditions).

It should be noted that the orientation of the methyl group of the T DNA base does not alter in the course of all reactions of conformational transitions. At this, the heterocycles of the DNA bases, capable for the out-of-plane bending^99–101^, stay planar.

So, obtained by us results launch the conception of the “mechanics” of the non-dissociative WC/rWC ↔ H/rH conformational transformations of the classical А∙Т DNA bps.

Of course, in the composition of DNA these conformational transitions represent a self-consistent transformation of the bps, the *anti* ↔ *syn* transition of A around the glycosidic bond (ΔΔG_TS_ = 3.4 kcal∙mol^−1^ at χ_TS_ = 121^◦^ for BI-conformer of the isolated 2′-deoxyadenosine^102^) and reorganization of stacking and hydratation^8^. Simple comparison of the energetics, determining these processes, clearly indicates that the first two of them plays a leading role. This fact gives hope that obtained in this paper data are closely related to the nature of the А∙Т(WC) ↔ А∙Т(H) thermal fluctuation process, which occurs in DNA^1–7^. This conclusion can be verified, applying the newest methods of *ab initio* dynamics for the short fragments of DNA.

## Conclusions

By applying developed by us novel ideas according the high-energetic conformers of the classical А∙Т DNA bps^11^, we offered novel non-dissociative mechanisms of the А∙Т(WC) ↔ А∙Т(H) and А∙Т(rWC) ↔ А∙Т(rH) conformational transitions, that do not require for their realization energy-consuming anisotropic rotation of the amino group of the A DNA base around the C6-N6 exocyclic bond. Figuratively speaking, at the transformation of the A base from the *anti-* to *syn-*conformation leading to the formation of the Hoogsteen А∙Т(H) and reverse Hoogsteen А∙Т(rH) bps, it dynamically relies as on the support on the T DNA base through the pyramidilized amino group of A, interacting with it in the TS region by one single (Т)N3H···N6(А) H-bond.

In the light of the obtained by us results, it could be suggested that the А∙Т(WC) ↔ А∙Т(H) conformational transition in DNA duplex, which was registered experimentally^1–7^, most likely occurs by the non-dissociative mechanism: A, rotating from the *anti-* to *syn-*configuration, interacts with T *via* the intermolecular H-bonds along the entire process of the conformational transformation.
